# Atypical appearance of elastofibroma dorsi at thoracotomy sites: a case series and review of the literature

**DOI:** 10.1259/bjrcr.20190112

**Published:** 2020-09-29

**Authors:** Francis Girvin, Syed Hoda, Stacy Kim

**Affiliations:** 1 Department of Radiology, NYU LangoneMedical Center, New York, United States; 2 Department of Pathology, NYU Langone Medical Center, New York, United States

## Abstract

Thus far, only a single case describing an atypical appearance of elastofibroma dorsi at a prior thoracotomy site has been published in the literature. We describe a series of three cases recently imaged at our institution with the same atypical appearance, in order to highlight and increase recognition of this more unusual morphology in post-operative patients.

## Introduction

Elastofibroma dorsi are benign reactive masses with characteristic mixed fatty and soft tissue morphology and subscapular location that are typically readily recognizable on ultrasound, CT and MRI of the chest. Widened intercostal spaces at thoracotomy sites, however, may result in elastofibroma formation, enlargement and medial herniation into the thoracic cavity. The resulting atypical appearance may present a diagnostic challenge on imaging, and may be mistaken for aggressive processes, precipitating unnecessary work-up or even resection.

## Cases

### Case 1

**Figure 1.**
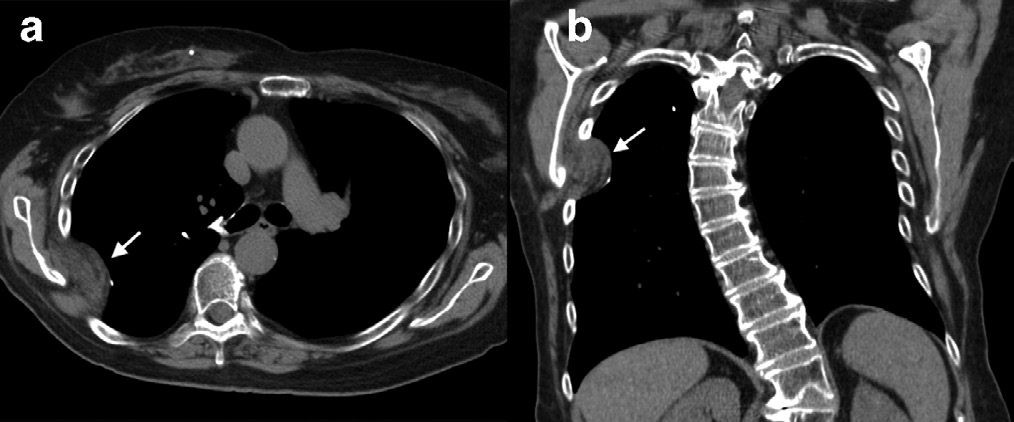
(a, b) Axial and coronal CT images in 79-year-old female (Case 1) showing a unilateral right-sided mixed soft tissue and fatty chest mass herniating medially through a widened rib interspace at a prior thoracotomy site.

**Figure 2.**
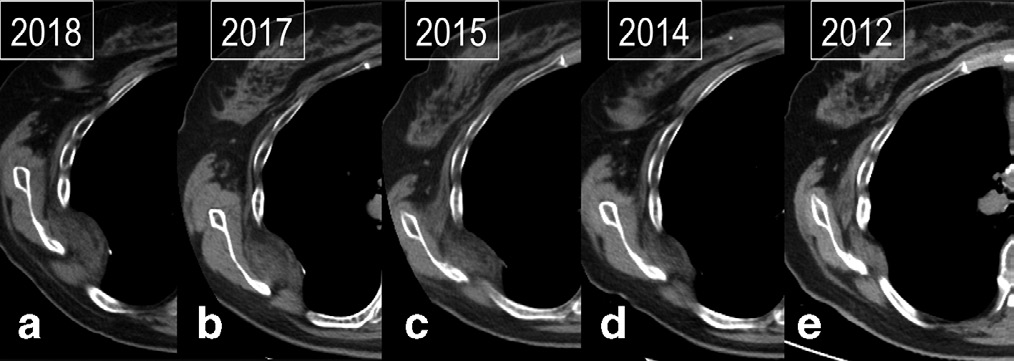
(a–e) Serial CT images showing progressive enlargement of chest wall mass over 6 years.

**Figure 3.**
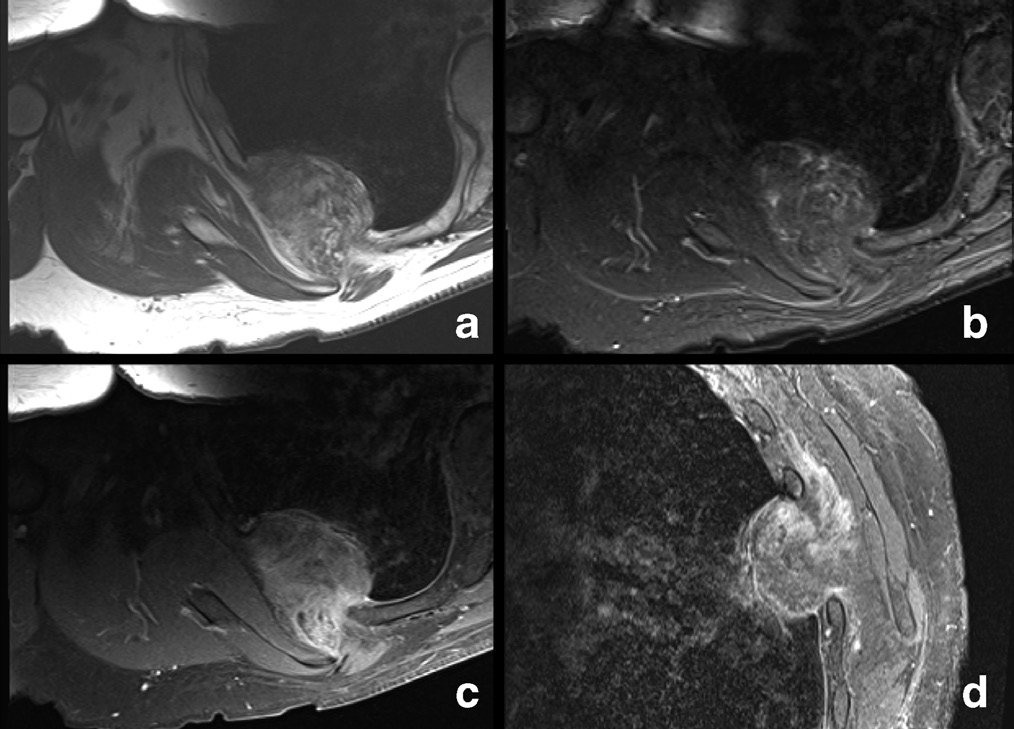
(a–d) 3a axial T1, 3b axial STIR, 3c axial T1 post-gadolinium, 3d Sagittal T1 post-gadolinium. Enhancing mixed soft tissue and fat signal intensity right chest wall mass. STIR, short tau inversion recovery.

**Figure 4.**
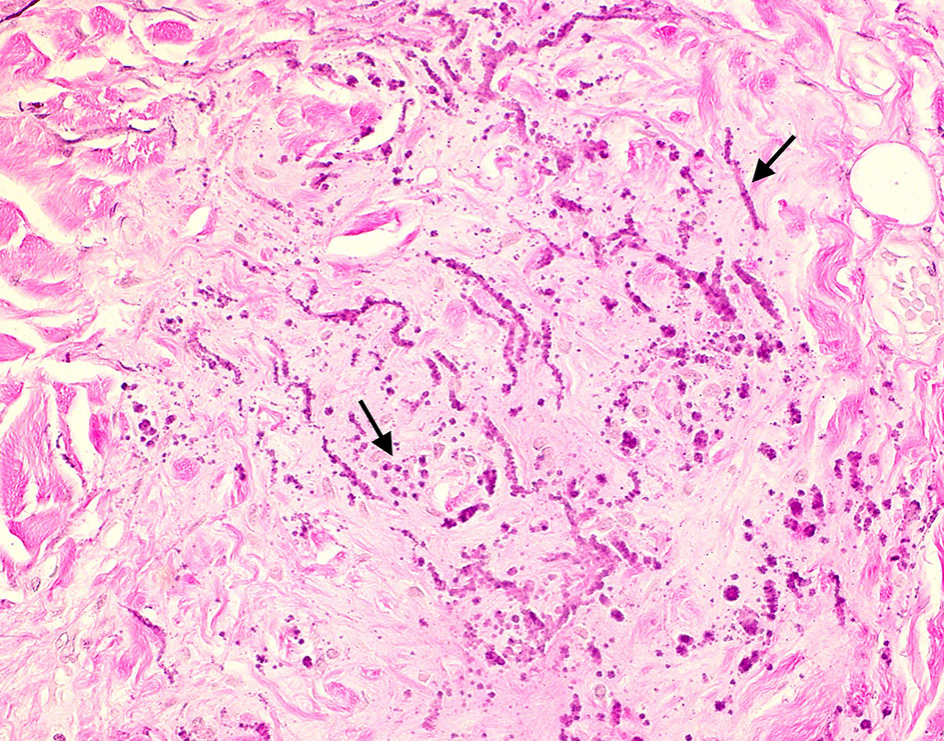
High magnification image of the specimen with elastic stain highlighting the abundant coarse elastic fibers.

A 79-year-old asymptomatic female with a history of right upper lobectomy 10 years earlier for a Stage 1A adenocarcinoma of the lung had a unilateral right chest wall mass noted on chest CTs performed at an outside institution (Figure 1A and B). The finding was not present on the pre-operative or earlier post-operative CTs, but developed 4 years following surgery and gradually increased in size on follow-up (Figure 2A, B, C, D and E). The outside scans were uploaded to our institution for interpretation and the mass interpreted as suspicious for liposarcoma, with biopsy and chest wall MRI advised. Avid gadolinium enhancement within the mass (Figure 3A, B, C and D) and enlargement from earlier post-operative CTs was again interpreted as concerning for sarcoma and elastofibroma was not considered in the differential diagnosis. The patient proceeded to biopsy on the basis of the MRI and CT interpretations, with pathology demonstrating an elastofibroma. Although asymptomatic, the patient was referred to cardiothoracic surgery, and a decision made to resect. Pathology of the resected lesion demonstrated a mixed fibrotic and fatty mass with prominent elastic fibers and areas of skeletal muscle, consistent with an elastofibroma (Figure 4). 2 weeks following resection, the patient re-presented with fevers, night sweats and chest wall pain, and a chest CT demonstrated a collection tracking along the chest wall graft. Aspiration of the collection demonstrated Gram-positive rods on microscopy, and the patient required a second thoracoscopy for chest wall graft revision given concerns for perigraft infection. The patient’s post-operative recovery has proved slow, with new restrictions in mobility related to back pain at the operative site and new depression, having previously been completely functional prior to any intervention for this incidental finding that was only detected on imaging.

### Case 2

**Figure 5.**
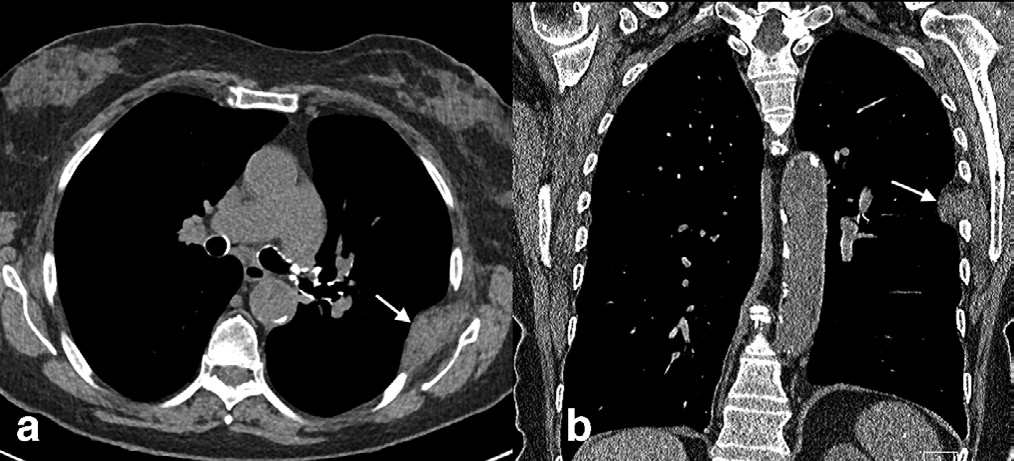
(a, b) Axial and coronal CT images in an 80-year-old female (Case 2) showing a unilateral left-sided mixed soft tissue and fatty mass herniating medially through a widened rib interspace at a prior thoracotomy site.

An 80-year-old asymptomatic female patient with history of left upper lobe wedge resection for atypical adenomatous hyperplasia 13 years earlier had a unilateral left-sided chest wall soft tissue mass noted on post-operative chest CTs (Figure 5A and B) performed for surveillance of groundglass nodules. The finding was not present on the pre-operative or earlier post-operative CTs, but developed 5 years following surgery and slightly enlarged on subsequent scans. Multiple chest CT reports raised fibrous tumor of the pleura or malignancy as possible etiologies, and the diagnosis of elastofibroma was not suggested. A biopsy was performed after recommendation by radiology, and demonstrated benign spindle cells and collagenized fibroadipose tissue, compatible with an elastofibroma.

### Case 3

**Figure 6.**
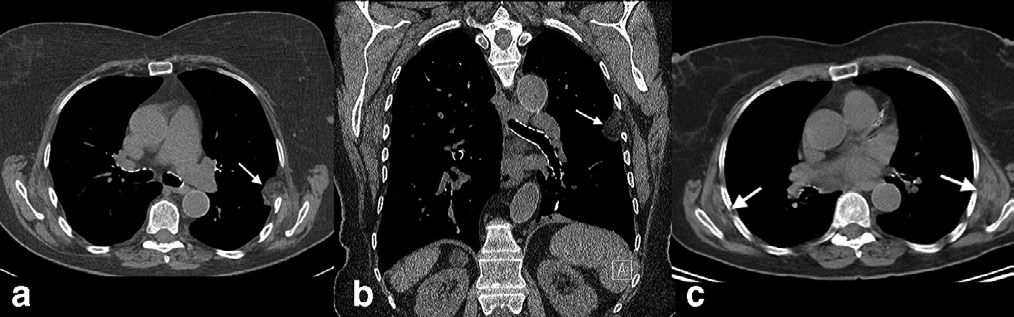
(a, b) Axial and coronal CT images in an 80-year-old female (Case 3) showing a left-sided mixed soft tissue and fatty mass herniating medially through a widened rib interspace at a prior thoracotomy site. Axial CT image in same patient prior to the left thoracotomy, showing bilateral (left greater than right) subscapular chest wall elastofibroma dorsi.

An 84-year-old asymptomatic female underwent a left lower lobe wedge resection for typical carcinoid tumor 9 years earlier. Post-operative chest CTs (Figure 6A and B) were initially interpreted as showing post-surgical chest wall fat herniation into the left thoracic cavity at the thoracotomy site; however, a subsequent review of the pre-operative CTs demonstrated bilateral elastofibroma dorsi (Figure 6C), confirming that the post-operative appearance reflected herniation of a pre-existing left-sided elastofibroma through a widened interspace at the thoracotomy site.

## Discussion

Elastofibroma dorsi are benign reactive masses composed of variable amounts of fibroblasts, collagen, elastin fibers and fat,^1^ thought to result from repetitive mechanical stresses on the chest wall soft tissues due to friction between the scapula and ribs. Lesions are often asymptomatic and only detected as an incidental finding on imaging, however, larger lesions may manifest as a chest wall mass on physical exam, and approximately 50% of patients may complain of a clunking sensation or local swelling on movement.^2^ Although initially described as rare, with a reported prevalence of 2% on one CT study,^3^ autopsy series have shown an incidence of up to 24% of females and 11% of males older than 55 years of age,^3^ and pre-elastofibroma changes (defined as a few or many degenerated elastin fibers) identified in up to 81%.^4^ The true incidence of elastofibroma may be underestimated on imaging, as lesions may be overlooked when small or symmetric, given the often similar morphologic appearances to normal chest wall soft tissues. Imaging findings are in general pathognomonic, showing a subscapular mass with variable mixed fatty and soft tissue on ultrasound, CT or MRI,^5^ with no need for biopsy for typical appearances, and surgery only reserved for symptomatic cases. The incidence of bilaterality has a wide reported range of 10–60%^6^ ; although a contralateral lesion effectively excludes a malignant process, a unilateral lesion with typical appearances should therefore not raise concern. In Case 1, the avid enhancement on MRI was interpreted as a suspicious finding, however, radiologists should be aware that highly variable enhancement patterns are recognized, including marked enhancement,^6^ and should not raise concern in a lesion with otherwise typical morphology and location for an elastofibroma. The healthcare costs and morbidity associated with the repeated chest CT follow-up, chest MRI, biopsy, two separate surgeries, inpatient stay, and post-operative recuperation for this particular case were not insignificant, for an incidental benign lesion in a previously asymptomatic elderly patient.

In two of our cases, the elastofibroma dorsi were not present pre-operatively, but developed at the widened rib interspace after an interval of several years following surgery, and subsequently enlarged at follow-up. Widened rib interspaces at thoracotomy sites may therefore have an etiologic role in both elastofibroma formation and enlargement, by creating a deeper pocket medial to the scapula with altered dynamic mechanical stresses on the chest wall tissues.

A literature search reveals only one other published case of an atypical presentation of an elastofibroma dorsi indenting into the thoracic cavity through a widened rib space following thoracotomy in a middle-aged female patient, involving a lesion that was already present pre-operatively.^7^ Iwata et al also published a case report of an enlarging, morphologically identical mass at a thoracotomy site in an elderly female patient, with pathology demonstrating muscle and connective tissue, and ascribed the finding to “an inverted intercostal hernia”^8^ ; however, as that entity has no other descriptions in the literature, and the history and imaging findings were identical to our three cases, it is the authors’ opinion that this was much more likely an unrecognized elastofibroma, lending further support that this atypical appearance provides a diagnostic challenge.

In conclusion, in addition to the already well-described imaging radiologic appearances of elastofibroma dorsi, lesions may also develop, enlarge or herniate medially at widened intercostal spaces following thoracotomy. The finding of three cases with identical CT appearances all imaged within the last year at our institution would support that this may be a less rare constellation of findings than already reported in the literature. Recognition of this more unusual appearance of elastofibroma dorsi by radiologists, clinicians and surgeons should obviate the need for other costly imaging tests, follow-up, biopsy or surgical resection in patients with this incidental benign finding.

## Learning points

1. Elastofibroma dorsi may develop, enlarge and herniate medially at widened rib spaces in post-thorcotomy patients, particularly in elderly females.
2. Avid gadolinium enhancement is one of several recognized enhancement patterns in elastofibroma dorsi and should not be interpreted as suspicious in a lesion with typical morphology and location.
3. Biopsy, follow-up and resection are typically not required for incidental, asymptomatic elastofibroma dorsi.
